# Multiple myeloma and farming. A systematic review of 30 years of research. Where next?

**DOI:** 10.1186/1745-6673-3-27

**Published:** 2008-11-17

**Authors:** Carla Perrotta, Anthony Staines, Pierlugi Cocco

**Affiliations:** 1 School of Public Health and Population Science, University College Dublin, Woodview House, Belfield, Dublin 4, Ireland; 2 School of Nursing, Dublin City University, Glanesvin, Dublin 9, Ireland; 3 Department of Public Health, Occupational Health Section, University of Cagliari, Italy

## Abstract

**Background:**

Multiple myeloma has been linked to farming for over thirty years. However, there is little clarity about the magnitude of the risk, nor about the specific agricultural exposures which contribute to the risk.

**Methods:**

We have carried out a systematic review of case-control studies of multiple myeloma published from 1970 to October 2007. Studies were identified through database searches and from references in the literature.

Studies reporting risk estimates from farming, agricultural exposures, and exposure to animals were identified, and details abstracted. The impact of study heterogeneity, publication bias, variation in methods of case identification and exposure ascertainment between studies were considered in analysis.

**Results:**

Case control studies showed a pooled odds ratio (OR) for working as a farmer of 1.39 95% CI 1.18 to 1.65. There was no graphic evidence of publication bias, for pesticide exposure 1.47; 95% 1.11 to 1.94, for DDT 2.19; CI 95% 1.30 to 2.95; for exposed to herbicides 1.69; 95 %CI 1.01 to 1.83. For working on a farm for more than ten years OR was 1.87; 95% CI 1.15 to 3.16.

**Conclusion:**

Farmers seem to have increase risk for MM. However, a major limitation of this analysis is the presence of significant heterogeneity across the studies and the evidence of publication bias in some models.

A pooled analysis using individual level data could provide more power and permit the harmonization of occupational and exposure coding data.

## Background

Multiple myeloma (MM) is a non-Hodgkin's lymphoma (NHL) with a number of distinctive clinical and biological features, which distinguish it from other members of the NHL family.

The disease is uncommon in younger people, but the incidence rises steeply from the age of 65. There is a marked male predominance. There is very substantial variation in incidence between different countries with the lowest incidence rates amongst in Asia and highest rates in wealthy countries, and especially in the black population in North America. [1,2]

Over the last thirty years, more than sixty studies have been carried out into the etiology of multiple myeloma. In contrast to the immense progress in understanding the biology of this disease, and in the development of new therapies, for most patients, little can be said about the possible causes. No useful public health interventions have been identified which might reduce the incidence of this disease, or the individual risk of this disease.

Most of the work on the etiology of MM focuses on understanding potential risks associated with long term environmental and job related exposures. Several occupations and exposures have been related to MM, notably working in agriculture, printing services and some specific chemical industries such as plastic and rubber.

We present a meta analysis of case control studies of occupation and MM. This first article reports our analyses of those studies reporting the effect of farming and other agricultural exposures. Our goal is to clarify what is presently known, and to provide a context for designing the next generation of epidemiological studies on myeloma.

### Farming and Multiple Myeloma

Farming has been consistently associated with an increased risk of MM since 1970 when Milham (1970) [3] reported a higher than expected number of MM deaths amongst American farmers. Khuder and Mutgi (1997)[4] published a meta analysis of farm employment and MM and assessed 32 case control and cohort studies done between 1981 and 1996.

The pooled analysis of the OR from individual papers (both case control and cohort studies) showed a relative risk of 1.23, with a 95% confidence interval (95% CI) of 1.14 to 1.32 for the association between MM and farming. A sub group analysis of female farmers reported a RR of 1.38; 95% CI 1.27 to 1.43. The sub group of thirteen cohort studies had a pooled RR of 1.13; 95% CI 1.09 to 1.17. However, they did not include an analysis of any particular agricultural exposures it combined case control and cohort studies and it did not report the presence of heterogeneity in those models. Since then, a number of important new studies have been conducted and further efforts have been made to identify possible agricultural exposures.

Our goal was to do a meta analysis of all case control studies on farming occupations published since 1970.

## Methods

### Search Strategy

PUBMED and the Cochrane Library were searched and we carried out further manual searching of reference lists of articles. The PUBMED search strategy used was (Myelom*$, Multiple Myeloma$, Plasmocytoma$, Plasmocytom$, or Plasmacytom$, Mieloma$, Lymphomas, Non- Hodking lymphomas .ti.ab.) AND (Job exposure or occupational exposure or Agricultural or farmers or farming or pesticides or glyphosate or dichloro-diphenyl-trichloroethane or insecticides or meat workers or occupational, or environmental exposure) ti.ab. We also explored the terms "controlled-study"/all sub headings (control or controls or controlled) with (trial* or study or studies) in title and abstracts and (evaluation or prospective*) with (trial* or study or studies) and (cohort or prospective studies). Studies that did not report separately on cases of MM were excluded as were papers with preliminary data.

There was no specific restriction on study inclusion based on quality scores. If two publications from the same study population were published we selected the most recent analysis for inclusion. Studies were searched from 1970 until October 2007. No language restriction was made.

### Analysis

For each case control study included we identified the job titles used for farmers and the nature and level of detail of agricultural exposures recorded. We extracted the odds ratio and associated confidence intervals for the following categories: "farmers", "agricultural or animal husbandry workers", "agricultural workers", pesticides exposure (ever/never), specific pesticides, and working as a farmer for more than ten years.

Consistency between studies was checked using the Cochran Q test under the null hypothesis that all studies have the same effect [5]. If heterogeneity was identified studies were excluded first according to their design (that is exposure extracted from death certificates or cancer registry data vs. personal interview), then year of publication (before 1999 vs., after 1999) and continent of origin (Europe vs. rest of the world).

Random effects models were used to estimate the pooled OR and the associated confidence interval for each specific exposure or job title. The adjusted estimate was included in the pooled analysis.

Funnel plots were drawn to evaluate potential publication bias [6]. All the analyses were done using Stata, version 9.

## Results

### Studies retrieved

Fifty five relevant studies were selected out of 530 cites obtained using this broad search strategy.

From the fifty five studies identified, 28 case control studies reported estimates for farming and/or agricultural related exposures. [3,7-32] (Additional file 1).

### Studies description

Researchers used a range of methods to identify cases. These included routinely collected death certificates, and death certificates from the American Cancer Society Prospective Study. In all the other studies, cases were reported to cancer registries or identified as incident cases in individual hospitals (additional file 1). Controls were matched by age and gender in all the studies. Reporting of estimates separately for women was done in three studies [7,13,30].

Occupational assessment was done using either job title or occupation as recorded on the death certificate [3,11,12,25,32], surveys [8], occupation registries [21,30]or using standardized interviews [3,7,9,10,13-20,22,24,26,28,29,31] Four studies [8,15,23,33] reported duration of occupation in categories.

Exposure assessment varied across these studies. Most used a detailed occupational history and obtained further information from job-specific questionnaires and some of them used hygienists to assess exposures from occupation history. [7,13,21,23,30] The specific exposures reported in the case control studies were pesticide exposure in general [7,8,16,21,24,29,30]; exposure to specific pesticides such as DDT [16,26], chlorophenols [16,26]; herbicides [27,29,33] and working with specific animals.

### Farming

**Figure 1:**
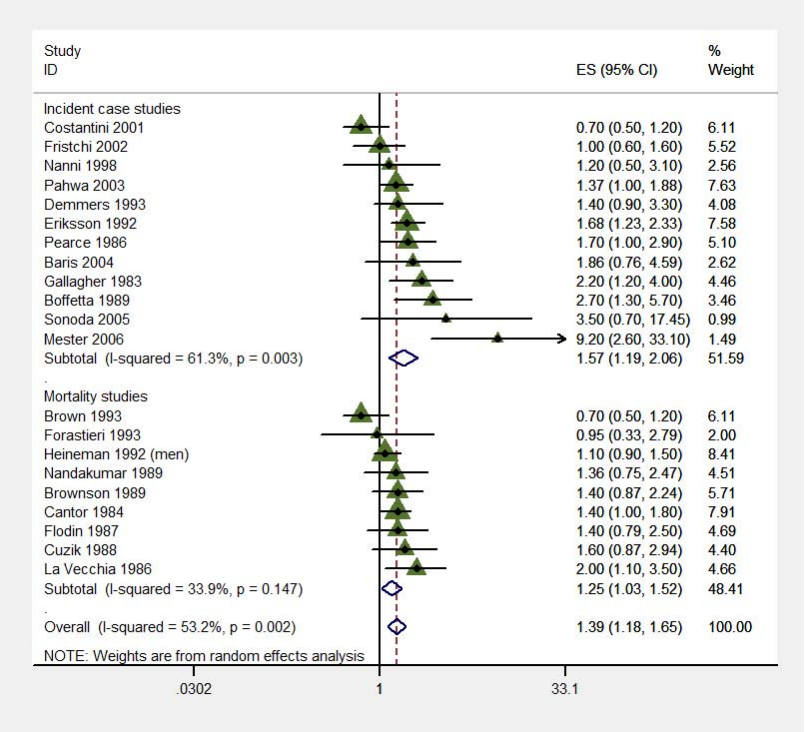
Individual and pooled OR for farming and Multiple Myeloma from published case control studies.

It was possible to extract the odds ratio and confidence interval from 21 case control studies. There was a 33% increase in the risk for ever working as a farmer (OR 1.39; 95% CI 1.18 to 1.65) (fig 1). There was significant heterogeneity in this model (p = 0.002). Heterogeneity persisted when analyzing European studies vs. rest of the world studies, and within Europe, Scandinavian countries vs. non-Scandinavian countries, and those studies done before and after 1999.

Sub group analysis was done according to the method used to ascertain occupation and according to co variables including obtaining the estimates.

The risk in those studies that ascertained occupation using death certificates was increased (OR 1.25; 95% CI 1.03 to 1.52) (fig 1) as well as those seven studies that adjusted for level of education (OR 1.28; 95% CI 1.02 to 1.62) None of this two models have presence of heterogeneity.

**Figure 2:**
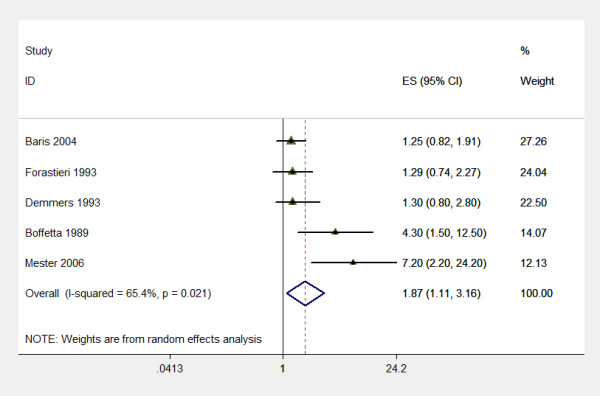
Farming and Multiple Myeloma, meta analysis of case control studies: Individual and pooled odds ratio, category "Working as a farmer for more than ten years".

We did a further subgroup analysis for ten or more years working on a farm (OR 1.87; 95% CI 1.11 to 3.16) (test for heterogeneity p = 0.021) (Figure 2).

### Pesticides

**Figure 3:**
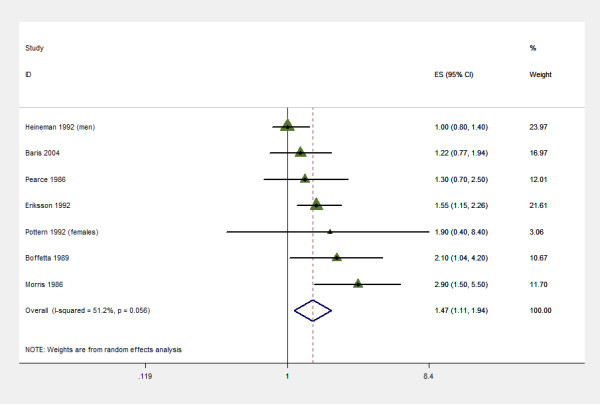
Farming and Multiple Myeloma, meta analysis of case control studies: individual and pooled estimates, category "ever exposed to pesticides".

Seven case control studies [7,8,16,21,24,29,30] reported pesticide exposure (analyzed here as ever or never occupationally exposed). Ever being exposed to pesticides had increase risk (OR 1.47; 95% CI 1.11 to 1.94, with evidence of heterogeneity and publication bias (test for heterogeneity p = 0.090). (Figure 3, additional file 3).

Few studies reported on specific pesticides groups. DDT exposure had an increase risk [16,26] (OR 2.19; 95% CI 1.30 to 2.95); herbicide exposures were also reported separately in three studies [7,27,29] with no increase in the risk (OR 0.97; 95% CI 0.68–1.38) (results not shown). Two compounds showed high estimates in individual studies: phenoxyacetics (OR 2.2; 95% CI 1.15–4.66 and chlorophenols (OR 2.4; 95% CI 1.0–5.9).

### Animal farming

Four studies reported OR for working with different animals [7,16,26,29].

The risk was increase for working in a farm with sheep (OR 1.71; 95% CI 1.25–2.33); [16,29]; horses (OR 1.72; 95% CI 1.26–2.37) [16,26];and, dairy cattle (OR 1.59; 95% CI 1.26–2.01) [7,16,26,29] (results not shown).

These results should be taken with caution as all of the models have graphic evidence of publication bias (results not shown).

## Discussion

There is consistent evidence from studies in many different countries over nearly thirty years for an association between working on a farm and multiple myeloma. A previous meta-analysis published in 1997 summarized the evidence at that time. [4]

Pooling data with the new case control studies further confirms the association between farming and MM (OR 1.39; 95% CI 1.18 to 1.65). The association is not strong but it is very consistent over many years in several different countries. However, the presence of heterogeneity was significant in most of the models. The main source of heterogeneity seems to be study design, adjustment for level of education in the individual studies estimates and the different farming techniques and pesticide use around the world and across the decades.

For a more specific study of 'agricultural exposures', there is now substantial published data on exposures to pesticides but not strong evidence on working with animals. Ever being exposed to pesticides have a 46% increase in risk. Exposure to DDT, chlorophenols and phenoxy-acetic acids were all associated with an excess risk in case-control studies of MM patients.

These results should be taken with caution as the presence of publication bias was significant in both models working with animals and exposure to pesticide.

Plotting the standard error of the OR over the OR gives an asymmetric figure suggesting reporting bias, as authors are much more likely to report exposures with statistically significant odds ratios.

It is possible that there are a number of unpublished negative studies, and that if these were available our conclusions would be modified.

A major limitation of this meta analysis is that we did not included cohort studies. However, pooling the cohort studies that were included in the Kruger meta analysis plus the cohort studies that were published after its publication [34,35] gave an relative risk of 1.12 (95%CI 1.09 to 1.16) with strong evidence of heterogeneity (p = 0.006) as well in the models.

The Agricultural Health Study; a large ongoing cohort study set in the US, has produced five publications so far on the risk of MM and specific pesticides. None of these associations has reached statistical significance, and it may be that more follow up time from these cohorts will be needed. As MM has a well defined pre malignant state (MGUS) the study of MGUS in agricultural cohorts could lead to interest results in the future.

All observational studies are subject to a range of methodological problems that are more severe, and far less tractable, than those affecting clinical trials. Methodological checklists for case-control study quality assessment have been developed, but these have yet to achieve widespread use [36]. Adoption of a formal meta-analytic approach, as done here, can be viewed as simply providing a convenient tool for summarizing the result of a large number of studies. It is, at least, useful.

Are there public health implications from our work? The hazards of farming both in developed and developing countries are well established. There is no current evidence that the individual risk of myeloma can be modified. It is possible that the very largest cohort studies will record farming practices in sufficient detail, and accrue enough cases of myeloma, to permit an effective analysis, but this will take many decades. Public health advice, while accepting the scanty evidence base, could probably focus first on minimizing the use of agro-chemicals, and then on the use of protective equipment by applicators.

This review shows the limitations of how observational studies report their results; as an example it would have been interesting to analyze the effect of exposure on females and males separately but it was not feasible as few studies reported estimates for gender categories.

Future systematic reviews will need to pool individual level data from as many studies as possible, which would permit a much more robust analysis, and the ability to adjust odds ratios for variables not considered in the original analysis as well as the possibility of harmonization of the occupational and exposure categories. Future case-control studies of myeloma will need to be an order of magnitude bigger than all current studies, and will need very refined exposure assessment if they are to contribute to progress.

## Conclusion

Farmers seem to have increase risk for MM. Pesticides rather than animal exposure seems to be a possible risk factor. However, a major limitation of this analysis is the presence of significant heterogeneity across the studies and the evidence of publication bias in some models. Level of education seems to be an important co variable and should be considered in studies analysing occupation.

A pooled analysis using individual level data could provide more power and permit the harmonization of occupational and exposure coding data.

## Abbreviations

MM: Multiple Myeloma; OR: odds ratio; 95%CI: 95% Confidence Interval

## Competing interests

The authors declare that they have no competing interests.

## Authors' contributions

CP has carried out the data extraction, performed the statistical analysis and drafted the manuscript, AS conceived the study and helped to draft the manuscript, PC participated in the writing on the manuscript.

## Supplementary Material

Additional file 1 **Table 1.** Farming and Multiple Myeloma, meta analysis of observational studies control studies: Description of case control studies Click here for file

Additional file 2 **figure 1.** Farming and Multiple Myeloma, meta analysis of case control studies. Funnel plot. Ever/never working as a farmer Click here for file

Additional file 3 **figure 2**. Farming and Multiple Myeloma, meta analysis of case control studies. Funnel Plot. Ever/never pesticide exposures. Click here for file
