# Supplementary material for: Multiple myeloma and farming. A systematic review of 30 years of research. Where next?
Source: J Occup Med Toxicol. 2008 Nov 17;3:27. doi: 10.1186/1745-6673-3-27 (PMC2628921; doi:10.1186/1745-6673-3-27)
Supplement: Additional file 1 — Table 1. Farming and Multiple Myeloma, meta analysis of observational studies control studies: Description of case control studies [file 1745-6673-3-27-S1.doc]

*Table1. Farming and Multiple Myeloma, meta analysis of observational studies control studies: Description of case control studies*

| Study | Years of observation | City/ Country | Cases Source  (Cases inclusion criteria) | Controls  Source  (matched criteria) |
| --- | --- | --- | --- | --- |
| Milham 1971 | 1950-1967 | Oregon State and Washington state, US. | Death certificate | Death certificate  (age, sex, month of death) |
| Gallagher 1983 | 1972-1978 | British Columbia, Canada | Incident and prevalent cases in a clinic. | Other cancer patients  (sex matched) |
| Cantor 1984 | 1968-1976 | Wisconsin state, US | Death Certificate  (White males) | Death certificate  (sex, age, year of death, region) |
| Pearce  1986 | 1977-1981 | New Zealand | Cancer Registry  (Males) | Cancer Registry  (sex, age, year entered at registry) |
| Nandakumar 1986 | 1975-1984 | Western Australia, Au | Cancer Registry  (Males) | Cancer Registry  (sex, age) |
| Flodin 1987 | 1971-1983 | Middle and Southeast Sweden | Hospital Labs | Population registers |
| Cuzick 1988 | 1978-1984 | England & Wales | Referral centres | Same hospital than the patient and GPs referrals  (sex , age) |
| Brownson 1988 | 1984-1988 | Missouri state , US | Cancer Registry  (White male) | All other cancer patients  (sex, age) |
| Boffetta 1989 | 1982 | US | American Cancer Prospective study (ACS) | ACS  (sex, age, ethnic origin) |
| La Vecchia 1989 | 1983-1988 | Milan | Referral centres | Hospitals |
| Pasqualetti 1990 | 1970-1988 | Italy | Hospital | Hospital controls  (sex, age) |
| Burmeister 1990 | 1964-1978 | Iowa state residents, US. | Death certificate  (White males) | Death certificate  (age, sex, year of death) |
| Eriksson  1992 | 1982-1986 | Northern Sweden | Swedish Cancer Registry | Population registry  (age, sex, county) |
| Heineman 1992 | 1970-1984 | Denmark | Danish Cancer Registry  (Males) | Population based controls  (age, sex, region) |
| Pottern 1992 | 1970-1984 | Denmark | Danish Cancer Registry  (Females) | Population based controls  (age, sex, region) |
| Brown 1993 | 1981-1984 | Iowa, US | Cancer registry  (white men) | Population based controls  (age, sex, vital status) |
| Franceschi 1993 | 1988-1991 | North east Italy | Hospitals | Hospital controls  (age, sex, region) |
| Demers 1993  and Morris 2006 | 1977-1981 | Counties in three US states | Cancer Registry | Population based controls  (age, sex, area) |
| Fritschi  1996 | 1979-1985 | Residents in Montreal | New histological confirmed case  (Males) | cancer patients and population controls  (age, sex, area) |
| Nanni 1998 | 1987-1990 | Forly Province, Northern Italy | Cancer Registry | Population registers  (age, sex) |
| Constantini 2000 | 1991-1993 | 12 areas from Italy | All incident cases from hospitals in the area | Population based controls  (age, sex, region) |
| Fritshi 2002 | 1994-1998 | 10 Canadian provinces, Canada | Cancer Registry | Population based controls  (age, sex) |
| Pahwa 2003 | 1971-1991 | Canada , six provinces | Cancer registry  (Males) | Population based controls  (sex, age, province) |
| Baris 2004 | 1986-1989 | Counties in Atlanta, New Jersey and Detroit, US | Cancer Registry | Population based controls  (age, race, sex, counties) |
| Sonoda 2005 | 2000-2004 | Japan | Cases in a referral hospital | Hospital controls  (age, sex) |
| Svec 2005 | 1984-1998 | 24 U States | Death certificates | Death certificate  (age, sex, race, region) |
| Mester 2006 | Non reported | Six regions of Germany | New cases in referral centres | Population based controls  (age, sex, region) |
