# Supplementary figures and images for: Multiple myeloma and farming. A systematic review of 30 years of research. Where next?
Source: J Occup Med Toxicol. 2008 Nov 17;3:27. doi: 10.1186/1745-6673-3-27 (PMC2628921; doi:10.1186/1745-6673-3-27)

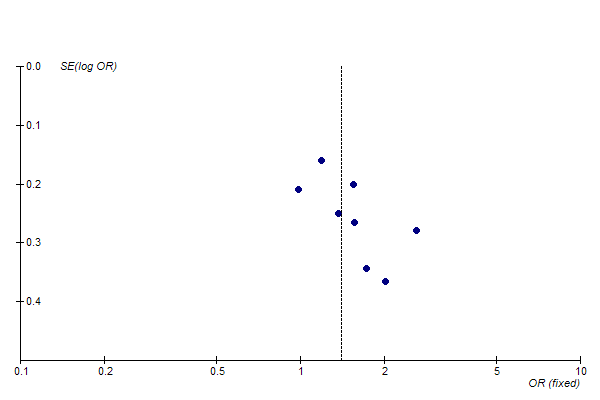

Supplement: Additional file 3 — figure 2. Farming and Multiple Myeloma, meta analysis of case control studies. Funnel Plot. Ever/never pesticide exposures. [file 1745-6673-3-27-S3.doc]
