# Supplementary material for: Multiple myeloma and farming. A systematic review of 30 years of research. Where next?
Source: J Occup Med Toxicol. 2008 Nov 17;3:27. doi: 10.1186/1745-6673-3-27 (PMC2628921; doi:10.1186/1745-6673-3-27)
Supplement: Supplementary file 3 [file 1745-6673-3-27-S2.doc]

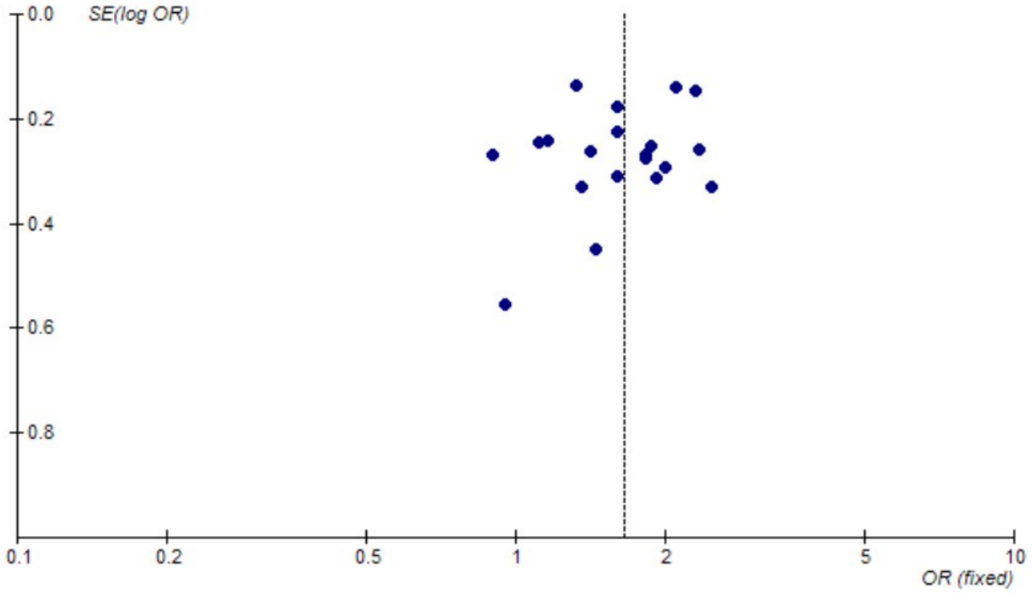

SE(log OR)
0.0
0.2
0.4
0.6
0.8
0.1
0.2
0.5
1
2
5
10
OR (fixed)
